# Knowledge, Attitudes, and Practices of nurses regarding blood culture collection

**DOI:** 10.1590/0034-7167-2023-0424

**Published:** 2025-01-13

**Authors:** Juliana Silva Ruiz, Oleci Pereira Frota, Marcos Antonio Ferreira

**Affiliations:** I Universidade Federal de Mato Grosso do Sul. Campo Grande, Mato Grosso do Sul, Brazil

**Keywords:** Blood Culture, Nursing Care, Blood Specimen Collection, Health Knowledge, Attitudes, Practice, Quality of Health Care, Cultivo de Sangre, Atención de Enfermería, Recolección de Muestras de Sangre, Conocimientos, Actitudes y Práctica en Salud, Calidad de la Atención de Salud

## Abstract

**Objectives:**

to investigate the knowledge, attitudes, and practices of nurses regarding blood culture collection.

**Methods:**

a cross-sectional study was conducted in five Brazilian public hospitals with 112 nurses. Data were collected using an adapted questionnaire and analyzed through descriptive and inferential statistics.

**Results:**

nurses who did not consider themselves capable of collecting blood cultures had a 72% lower chance of performing the collection at the recommended site and an 83% lower chance of using the same needle for blood inoculation into the vials. Nurses working in the emergency department had a 75% lower chance of knowing the international benchmark for blood culture contamination rates, and those with less than 5 years in the position decreased their chance of accuracy in this matter by 79%.

**Conclusions:**

there are gaps in the knowledge, attitudes, and practices of nurses regarding blood culture collection. Standardization of the technique, periodic education, supervision and guidance of the collection team, and process auditing are recommended coping strategies.

## RESUMEN

**Objetivos:**

investigar los conocimientos, actitudes y prácticas de los enfermeros sobre la recolección de hemocultivos.

**Métodos:**

estudio transversal realizado en cinco hospitales públicos brasileños, con 112 enfermeros. Los datos fueron recopilados mediante un cuestionario adaptado y analizados mediante estadísticas descriptivas e inferenciales.

**Resultados:**

los enfermeros que no se consideraron capaces de recolectar hemocultivos tuvieron un 72% menos de probabilidades de realizar la recolección en el sitio recomendado y un 83% menos de probabilidades de utilizar correctamente la misma aguja de punción para inocular la sangre en los frascos. Los enfermeros que trabajan en la atención de urgencias tuvieron un 75% menos de probabilidades de conocer el punto de referencia internacional para las tasas de contaminación de hemocultivos, y aquellos con menos de 5 años en el cargo disminuyeron su probabilidad de precisión en este asunto en un 79%.

**Conclusiones:**

existen brechas en los conocimientos, actitudes y prácticas de los enfermeros sobre la recolección de hemocultivos. La estandarización de la técnica, la educación periódica, la supervisión y orientación del equipo de recolección, y la auditoría de los procesos son estrategias de afrontamiento recomendadas.

## INTRODUCTION

The blood culture is the most commonly used laboratory test in clinical practice for investigating suspected bloodstream infections (BSIs). The procedure involves collecting and inoculating a blood sample into a blood culture bottle containing optimal conditions for microbial growth. This test is crucial for identifying the pathogen causing the infection and its susceptibility profile to antimicrobials, enabling the safe management and treatment of BSIs^(1,2)^.

The isolation of microorganisms that are not pathogenic to the patient, such as those from skin microbiota, equipment, environmental surfaces, or the hands of the professional performing the collection, constitutes sample contamination^(1,3)^. Consequently, false-positive results can occur, leading to harm to the patient and the healthcare facility. This harm may include increased hospital stays, unnecessary additional interventions, patient and family distress, emergence of multidrug-resistant microorganisms (MDROs), unnecessary use of human and material resources, and burden on the healthcare system^(4)^.

Blood culture contamination rates provide an important metric regarding the quality of services provided and should be kept as low as possible^(4)^. However, several studies^(5,6,7,8,9)^ report high contamination rates, indicating that maintaining blood culture contamination rates below the internationally recommended benchmark of 3% by the American Society of Microbiology remains a longstanding challenge for healthcare facilities^(10)^.

Blood culture collection is a critical step and a frequent practice in the daily routine of nursing. Implementing collection protocols and team education contributes to reducing contamination rates^(11)^. Thus, various preventive strategies have been tested for decision-making and scientifically grounded protocol construction, including antiseptic techniques^(12)^, staffing responsible for collection^(13,14)^, antiseptic solutions^(15,16,17)^, training of collection teams^(5)^, and restricting sample collection to a trained and dedicated team for this purpose^(9)^, among others.

However, the literature lacks studies that evaluate the level of knowledge, attitudes, and practices of professionals who commonly collect blood for culture, particularly nursing staff. Few studies have been conducted on this subject^(18,19)^, with only one involving nurses^(20)^, and all point out, in addition to the knowledge gap, the need for team training to prevent practices and erroneous results that can influence the quality of care provided.

## OBJECTIVES

To investigate nurses’ knowledge, attitudes, and practices regarding blood culture collection.

## METHODS

### Ethical Aspects

The study adhered to national and international ethical guidelines and received approval from the Research Ethics Committee of the Federal University of Mato Grosso do Sul. The approval documentation is appended to this submission. Nurses who consented to participate in the study provided their signatures on the Informed Consent Form (ICF).

### Study Design, Setting, and Period

This study is a cross-sectional investigation conducted through a Knowledge, Attitudes, and Practices (KAP) survey, guided by the STROBE tool. It was carried out in the Emergency Medical Services (EMS) and Intensive Care Unit (ICU) departments of five large public teaching hospitals in the state of Mato Grosso do Sul: three situated in the capital city of Campo Grande and two in the state’s interior. Data collection occurred monthly during the first semester of 2022.

### Population, Inclusion and Exclusion Criteria

All nurses assigned to the investigated units were included, while those on leave or vacation and those who declined to respond to the questionnaire on at least three consecutive occasions were excluded. Nurses working in multiple institutions included in this study were counted only once.

### Study Protocol

Data were gathered using an adapted instrument^(20)^ comprising 25 questions divided into two sections: (i) sociodemographic and professional variables and (ii) KAP components. The latter contained nine objective questions with short responses (yes, no, or I don’t know) and pre-established options. The average completion time for the questionnaire was 10 minutes.

To assess the research’s feasibility and any potential interpretation difficulties with the questions, a pilot test was conducted with nurses from the Coronary Care Unit of one hospital before data collection commenced. Following the pre-test, it was decided to include the question: “Is blood culture collection part of your job routine?”. No further modifications were made to the instrument after this adjustment.

Professionals meeting the inclusion criteria and consenting to participate in the study by reading and signing the informed consent form were invited to join as volunteers. Data collection took place on-site, and participants were prohibited from consulting any information sources – including the internet, books, manuals, or colleagues. The researcher ensured the complete survey was filled out without assistance. To ensure reliable data, there was no time limit for completing the questionnaire.

### Analysis of Results and Statistics

The data underwent analysis using descriptive and inferential statistics in the open-source statistical software R, version 4.2.0. To examine the association of sociodemographic variables (sex, age, level of education) and professional variables (years of experience in the profession, years of service in the unit, and work shift) with the KAP components, either the Chi-Square Test or Fisher’s Exact Test was employed. The Odds Ratio (OR), with a 95% confidence interval (CI), was utilized to gauge the magnitude of associations. The Kolmogorov-Smirnov Test was applied to assess data normality. A significance level of 5% was set for all statistical tests conducted.

## RESULTS

**Figure 1:**
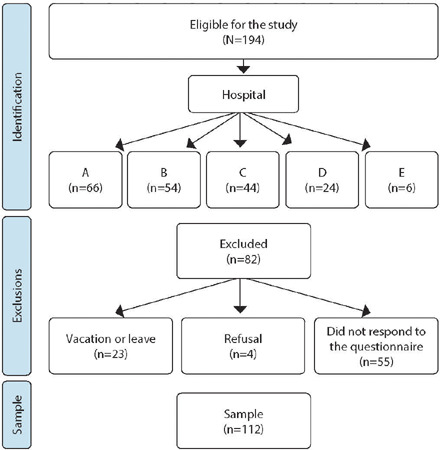
Flowchart depicting the sample selection process for the study, Campo Grande, Mato Grosso do Sul, Brazil, 2022

A total of 194 nurses were eligible for the study, of whom 82 (42.3%) were excluded: 23 (28%) due to being on vacation, leave, or absent, 4 (4.9%) for refusing to participate in the study, and 55 (67.1%) for being absent from their work sectors or refusing to respond to the questionnaire (Figure 1). Therefore, the sample consisted of 112 (57.7%) nurses, whose characteristics are presented in Table 1.

**Table 1:** Sociodemographic and occupational characteristics of nurses from three hospitals in the capital and two hospitals from the interior, (N=112), Campo Grande, Mato Grosso do Sul, Brazil, 2022

Variable	n	%
Sex		
Female	83	74,11
Male	29	25,89
Age, years (M±SD)	35,84±7,27	
≤ 35	48	54,55
> 35	40	45,45
Education		
Specialization in ICU or EMS	71	63,38
Specialization (except ICU and EMS)	37	33,04
Master's degree	2	1,79
Doctorate	2	1,79
Hospital		
A	38	33,93
B	29	25,89
C	24	21,43
D	17	15,18
E	4	3,57
Department		
EMS	45	40,18
ICU	67	59,82
Years of nursing education (M±SD)	10±5,55	
≤ 10	60	53,57
> 10	52	46,43
Years in current position years (M±SD)	6,50±5,33	
≤ 7	61	54,46
> 7	51	45,54
Years of experience in ICU or EMS, years (M±SD)	4,50±4,46	
≤ 5	66	58,93
> 5	46	41,07
Shift		
Morning	39	34,82
Afternoon	29	25,89
Night	44	39,29
Is blood culture collection part of your job routine?		
No	42	37,50
Yes	70	62,50
Have you received training for blood culture collection in this service?		
No	55	49,11
Yes	53	47,32
Don't remembre	4	3,57
Do you consider yourself capable of properly collecting blood cultures?		
No	26	23,21
Yes	86	76,79
Would you like to receive training to collect blood cultures?		
No	9	8,04
Yes	103	91,96
Who usually collects blood samples for blood culture tests in your department?		
Nurse	58	51,79
Laboratory technician	53	47,32
Nursing assistant	1	0,89
Does your hospital have a standard operating procedure for blood collection for culture?		
Yes	64	57,14
Don't know	48	42,86

*M – Média; DP – Desvio Padrão; UTI – Unidade de Terapia Intensiva; PAM – Pronto Atendimento Médico.*

The majority of nurses are female (74.11%) and hold specializations in ICU or EMS (63.38%). On average, they have 10 years of nursing education, with 53.57% having 10 years or less. The average duration in the profession is 6.50 years, with 54.46% working for up to 7 years. Most work in ICU (59.82%) for approximately 4.50 years in this department. Regarding blood culture collection, 62.50% report it as part of their routine work, 47.32% have received training for it, and 76.79% consider themselves adequately trained for proper collection. Moreover, 91.96% express interest in receiving additional training. As for standard operating procedures, 57.14% of nurses state that their hospital has them, while 42.86% do not have this information available (Table 1).

It was observed that nurses are the professionals who most often perform blood culture collections (51.8%). The majority performs a new vascular puncture when collecting blood samples for culture (68.7%) and uses 70% alcohol for both site antisepsis (74.1%) and disinfection of the tops of the bottles (89.3%).

There was a predominance of the clean/aseptic technique for collection (68.7%) compared to the sterile technique (29.5%), and the volume of 10 ml was the most indicated (58.9%) by nurses, although 66.1% did not know the percentage of the bottle corresponding to the ideal volume of blood to be collected. Table 2 presents the frequency of responses for the KAP components.

**Table 2:** Distribution of responses according to knowledge, attitudes, and practices of nurses from three hospitals in the capital and two hospitals from the interior regarding blood culture collection, (N=112), Campo Grande, Mato Grosso do Sul, Brazil, 2022

Questions and Answers	n	%
Practices		
From which site do you typically collect blood for blood culture?		
Peripheral venous access previously installed	7	6.25
Central venous access	26	23.21
New vascular puncture	77	68.75
Various	2	1.79
Which of the following solutions do you use to perform site antisepsis for blood culture collection?	0	
70% alcohol	83	74.11
Chlorhexidine solution	29	25.89
Which of the following solutions do you use to disinfect the tops of blood culture bottles?		
70% alcohol	100	89.29
Chlorhexidine solution	12	10.71
Attitudes		
How many milliliters of blood do you collect for the blood culture test?		
1 ml	1	0.89
5 ml	22	19.64
10 ml	66	58.93
20 ml	18	16.07
Other	5	4.46
What technique do you use to collect the samples?		
Sterile technique	33	29.46
Clean/aseptic technique	77	68.75
Don't know	2	1.79
Knowledge		
Which of the following alternatives is the blood culture contamination rate at your hospital?		
0-3%	4	3.57
3-5%	2	1.79
5-10%	5	4.46
>10%	2	1.79
Don't know	99	88.39
What is the internationally recommended value for the blood culture contamination rate?		
0%	4	3.57
<3%	18	16.07
<5%	9	8.04
<7%	1	0.89
Don't know	80	71.43
What percentage of the total volume of the collection bottle corresponds to the ideal volume of blood to be collected?		
5%	2	1.79
10%	17	15.18
15%	1	0.89
20%	15	13.39
Don't know	74	66.07
Other response	3	2.68
Is needle exchange between the collection puncture and the blood distribution in the blood culture bottle recommended?		
Yes	45	40.18
No	31	27.68
Don't know	36	32.14

The questions from Table 2 were correlated with the participants’ sociodemographic and occupational variables, and statistically significant associations are outlined in Table 3. It was discovered that nurses who do not consider themselves capable of collecting blood cultures were 72% less likely to perform the collection at the recommended site (OR: 0.28; 95% CI: 0.11-0.69) and 83% less likely to use the same needle for vascular puncture and blood inoculation into the blood culture bottle (OR: 0.07; 95% CI: 0.01-0.58). Concerning the internationally accepted benchmark of 3% for blood culture contamination rate ^(10)^, the likelihood of nurses in EMS compared to their ICU counterparts (OR: 0.25; 95% CI: 0.07-0.91); and (ii) 79% lower for those with less than 5 years in the profession compared to the longer-serving group (OR: 0.21; 95% CI: 0.07-0.63). Additionally, nurses working the morning shift had a higher percentage of correct answers regarding the benchmark compared to those working other shifts (p=0.008).

**Table 3:** Association of nurses’ knowledge, attitudes, and practices with sociodemographic and occupational data

Variável	Frequency (n) and percentage (%) of correct answers to the questions					
	Question 1		Question 2		Question 3	
	n(%)	*p* value	n(%)	*p* value	n(%)	*p* value
Sector						
EMS	29(64.4)	0.420	3(6.7)	0.026^†^	13(28.9)	0.815
ICU	48(71.6)		15(22.4)		18(26.9)	
Time of experience in EMS or ICU						
≤ 5 years	43(65.1)	0.325	5(7.6)	0.003^‡^	15(22.7)	0.161
> 5 years	34(73.9)		13(28.3)		16(34.8)	
Work shift						
Morning	26(66.7)	0.629	12(30.8)	0.008	9(23.1)	0.674
Afternoon	22(75.9)		2(6.9)		8(27.6)	
Night	29(65.9)		4(9.1)		14(31.8)	
Do you consider yourself adequately trained to collect blood cultures?						
No	12(46.2)	0.005*	2(7.7)	0.235	1(3.8)	0.002^§^
Sim	65(75.6)		16(18.6)		30(34.9)	

Question 1: “Where do you typically collect blood for blood culture?”; Question 2: “What is the internationally recommended value for the blood culture contamination rate?”; Question 3: “Is needle exchange between the collection puncture and the blood distribution in the blood culture bottle recommended?”; *OR: 0.28 [95% CI: 0.11-0.69]; ^†^OR: 0.25 [95% CI: 0.07-0.91]; ^‡^OR: 0.21 [95% CI: 0.07-0.63]; ^§^OR: 0.07 [95% CI: 0.01-0.58].

## DISCUSSION

This study represents the first investigation into the KAP of nurses working in ICU and EMS regarding blood culture collection in Brazil. Its findings significantly contribute to advancing our understanding by shedding light on the knowledge, attitudes, and practices of nurses in large teaching hospitals regarding blood culture collection. Through clinical practice observation, it becomes evident that nurses hold a pivotal role in blood culture sample collection. They not only execute the necessary technical and scientific competencies required by the procedure but also bear the responsibility of ensuring its safe and proper implementation within a hospital environment.

### Knowledge about blood culture collection

Despite being teaching hospitals, a lack of knowledge among professionals about the blood culture collection procedure was identified, which can be explained by insufficient training and education of the professionals to perform the procedure, as a significant portion (49.1%) did not receive training on-site. According to the World Health Organization, education and training are necessary for all personnel involved in blood sampling, including understanding anatomy, awareness of blood exposure risks, and the consequences of deficiencies in infection prevention and control^(21)^.

The majority of nurses were unaware of the blood culture contamination rate at their hospital (81.4%), the internationally recommended benchmark for blood culture contamination (71.4%), or the percentage of the total bottle volume corresponding to the ideal blood volume to be collected (66.1%). This finding is possibly supported by the lack of training provided to professionals and the absence of monitoring of care indicators.

Implementing training strategies for the collection team incurs immediate costs to institutions due to the need for ongoing education and investment in team training, which justifies why most nurses have not received training on-site. However, the benefits generated by qualifying and training teams reduce future costs, which can be even more burdensome than those incurred with training.

It was revealed that nurses working in the ICU with over 5 years of experience achieved higher accuracy regarding the internationally accepted value of 3% for the blood culture contamination rate. This finding may be justified by the ICU being a sector where hospital infections are more concerning and discussed. Nurses with more work experience have been exposed to more situations where they had to collect blood for culture and also have more contact with hospital infection prevention policies and bundles, such as Standard Operating Procedures (SOPs), although 43% of professionals are unaware of the existence of an SOP on blood culture collection in their hospitals.

Nurses working the morning shift had a higher percentage of correct answers compared to those on other shifts. Typically, sample collection for laboratory tests occurs predominantly in the morning period. Thus, professionals working in the morning are more accustomed to collecting biological material for tests, including blood cultures. As these nurses perform the procedure more often, they refine their collection technique and incorporate knowledge of the main guidelines, which may explain their higher accuracy.

Professionals who consider themselves capable of collecting samples had higher percentages of correct answers when questioned about the recommended puncture site and the need to change the needle between puncture and blood inoculation in blood culture bottles. It is inferred that nurses who consider themselves capable indeed have more knowledge, which generates self-confidence and security. Self-confidence, while not a determinant of knowledge, is an important attribute for technical-operational assertiveness. A study conducted with nursing undergraduates in Turkey showed that assertiveness and self-confidence are intrinsically associated^(22)^. These findings underscore the central role of knowledge in health indicators.

### Attitudes towards blood culture collection

The most commonly used technique by nurses for sample collection was the aseptic one (68.7%), justified by its practicality, low cost, and preference of professionals. A clinical trial^(11)^ concluded that more important than the technique used – aseptic or sterile – is the aseptic rigor in obtaining samples, as well as the application of collection protocols and the qualification of professionals performing it. In this KAP study, it was evidenced that the majority of nurses (57.1%) are aware of the institution’s SOP, but lack training and qualification.

Regarding nurses’attitudes during blood collection for culture, 58.9% aspirate 10ml for each blood culture bottle. This attitude may be anchored in the standardization of institutions regarding the procedure, as most professionals claim to be aware of the SOP of the place where they work. The *Agência Nacional de Vigilância Sanitária* (Anvisa) recommends that the amount of blood inoculated in each bottle be 10ml and argues that collecting the correct amount increases the chance of pathogen isolation by 15%^(23)^.

### Practices regarding blood culture collection

The majority of nurses (68.7%) perform a new vascular puncture, arterial, or venous, for sample collection. However, a considerable proportion of professionals still obtain blood from previously installed central (23.2%) and peripheral (6.2%) catheters. This practice is justified by the ease of obtaining samples, as a new vascular puncture requires more time from the professional. Additionally, there are often factors that hinder this new puncture, such as clinical condition, nutritional status, and scarcity of accessible vascular network, among others, which are further accentuated in more severe patients, who represent the majority profile of patients attended by the nurses participating in this study.

The most commonly used solution for both puncture site antisepsis (74.1%) and rubber stopper disinfection of blood culture bottles (89.3%) was 70% alcohol. Besides being efficient, this antiseptic is less costly compared to others commonly used in hospitals, such as alcoholic chlorhexidine, which probably explains its availability in institutions. A Randomized Clinical Trial conducted at a university medical center in Israel showed that both 70% alcohol and alcoholic solutions with 2% chlorhexidine gluconate and non-alcoholic solutions were able to reduce blood culture contamination^(15)^. However, a systematic review with meta-analysis demonstrated that alcohol-containing products are superior to alcohol-free products for blood culture collection due to alcohol’s immediate antiseptic action and its quick drying in up to 30 seconds^(16)^. The use of antiseptics is still controversial in the literature and lacks consensus on which solution is most effective in reducing contamination rates.

### Study limitations

The low adherence of professionals in responding to the questionnaire and its administration during routine service may have limited the results obtained, given that nurses completed it during their shifts. Another limitation to consider is the scarcity of literature and the lack of studies employing the KAP methodology on the addressed topic. Lastly, the cross-sectional nature of the study precludes the establishment of cause-and-effect relationships between the variables investigated.

### Contributions to Nursing and/or Health

This study not only furnishes valuable information about the practice of blood culture collection but also makes significant contributions to nursing and public health. By pinpointing critical points and knowledge gaps among nurses, this study underscores specific areas necessitating educational intervention and quality improvement programs. Implementing educational and training strategies based on the findings of this study could lead to substantial enhancements in blood culture collection practices, thereby aiding in the reduction of sample contamination and minimizing false-positive results.

## CONCLUSIONS

The findings of this research underscore the prevalence of nurse phlebotomists, the utilization of 70% alcohol among antiseptics, and the preference for the aseptic technique over the sterile one. Deficiencies were noted in the knowledge, attitudes, and practices of nurses in safely collecting blood culture samples. Nurses who perceive themselves as incapable of collecting blood cultures and have less than five years of experience in the profession exhibited unfavorable associations in the KAP questions. Addressing these challenges involves ongoing training and team qualification through continuing education, alongside the implementation of standardized collection protocols. Vigilance over processes and outcomes, as well as consistent supervision, guidance, and auditing of professionals regarding the correct execution of the procedure, are imperative. Subsequent research may evaluate the impact of these interventions.

## References

[1] Dawson S (2014). Blood culture contaminants. J Hosp Infect.

[2] Ombelet S, Barbé B, Affolabi D, Ronat JB, Lompo P, Lunguya O (2019). Best practices of blood cultures in lowand middle-income countries. Front Med (Lausanne).

[3] Bool M, Barton MJ, Zimmerman PA (2020). Blood culture contamination in the emergency department: an integrative review of strategies to prevent blood culture contamination. Australas Emerg Care.

[4] Hughes JA, Cabilan CJ, Williams J, Ray M, Coyer F (2018). The effectiveness of interventions to reduce peripheral blood culture contamination in acute care: a systematic review protocol. Syst Rev.

[5] Bowen CM, Coleman T, Cunningham D (2016). Reducing blood culture contaminations in the emergency department: it takes a team. J Emerg Nurs.

[6] Rupp ME, Cavalieri RJ, Marolf C, Lyden E (2017). Reduction in blood culture contamination through use of initial specimen diversion device. Clin Infect Dis.

[7] Ryan C (2017). Implementation of the theory of planned behavior to promote compliance with a chlorhexidine gluconate protocol. J Assoc Vasc Access.

[8] Self WH, Mickanin J, Grijalva CG, Grant FH, Henderson MC, Corley G (2014). Reducing blood culture contamination in community hospital emergency departments: a multicenter evaluation of a quality improvement intervention. Acad Emerg Med.

[9] Denno J, Gannon M (2013). Practical steps to lower blood culture contamination rates in the emergency department. J Emerg Nurs.

[10] Thomas S, Cheesbrough J, Plumb S, Bolton L, Wilkinson P, Walmsley J (2011). Impact of a blood culture collection kit on the quality of blood culture sampling: fear and the law of unintended consequences. J Hosp Infect.

[11] Frota OP, Silva RM, Ruiz JS, Ferreira-Júnior MA, Hermann PRS (2022). Impact of sterile gloves on bloodculture contamination rates: a randomized clinical trial. Am J Infect Control.

[12] Self WH, Speroff T, Grijalva CG, McNaughton CD, Ashburn J, Liu D (2013). Reducing blood culture contamination in the emergency department: an interrupted time series quality improvement study. Acad Emerg Med.

[13] Gander RM, Byrd L, DeCrescenzo M, Hirany S, Bowen M, Baughman J (2009). Impact of blood cultures drawn by phlebotomy on contamination rates and health care costs in a hospital emergency department. J Clin Microbiol.

[14] Al-Hamad A, Al-Ibrahim M, Alhajhouj E, Al-Alshaikh Jaffer W, Altowaileb J, Alfaraj H (2016). Nurses’ competency in drawing blood cultures and educational intervention to reduce the contamination rate. J Infect Public Health.

[15] Lalezari A, Cohen MJ, Svinik O, Tel-Zur O, Sinvani S, Al-Dayem YA (2020). A simplified blood culture sampling protocol for reducing contamination and costs: a randomized controlled trial. Clin Microbiol Infect.

[16] Maiwald M, Chan ES (2012). The forgotten role of alcohol: a systematic review and meta-analysis of the clinical efficacy and perceived role of chlorhexidine in skin antisepsis. PLoS One.

[17] O’Connor C, Philip RK, Powell J, Slevin B, Quinn C, Power L (2016). Combined education and skin antisepsis intervention for persistently high blood-culture contamination rates in neonatal intensive care. J Hosp Infect.

[18] Raupach-Rosin H, Duddeck A, Gehrlich M, Helmke C, Huebner J, Pletz MW (2017). Deficits in knowledge, attitude, and practice towards blood culture sampling: results of a nationwide mixed-methods study among inpatient care physicians in Germany. Infection.

[19] Yalçinkaya R, Öz FN, Erdoğan G, Kaman A, Aydın Teke T, Yaşar Durmuş S (2021). Turkish pediatric residents’ knowledge, perceptions, and practices of blood culture sampling. Arch Pediatr.

[20] Nair A, Elliott SP, Al Mohajer M (2017). Knowledge, attitude, and practice of blood culture contamination: A multicenter study. Am J Infect Control.

[21] Cadman H, Khamassi S (2014). Diretrizes da OMS para a tiragem de sangue: boas práticas em flebotomia [Internet].

[22] İlhan N, Sukut Ö, Akhan LU, Batmaz M (2016). The effect of nurse education on the self-esteem and assertiveness of nursing students: a four-year longitudinal study. Nurse Educ Today.

[23] Agência Nacional De Vigilância Sanitária (Anvisa) (2013). Microbiologia Clínica para o Controle de Infecção Relacionada à Assistência à Saúde. Módulo 4: Procedimentos Laboratoriais: da requisição do exame à análise microbiológica e laudo final[Internet].

