# Supplementary material for: Knowledge, Attitudes, and Practices of nurses regarding blood culture collection
Source: Rev Bras Enferm. 2025 Jan 13;77(6):e20230424. doi: 10.1590/0034-7167-2023-0424 (PMC11731844; doi:10.1590/0034-7167-2023-0424)
Supplement: Translation

# Translation: Conhecimentos, Atitudes e Práticas dos enfermeiros sobre colheita de hemocultura

**DOI:** 10.1590/0034-7167-2023-0424pt

**Authors:** Juliana Silva Ruiz, Oleci Pereira Frota, Marcos Antonio Ferreira

**Affiliations:** I Universidade Federal de Mato Grosso do Sul. Campo Grande, Mato Grosso do Sul, Brasil

**Keywords:** Hemocultura, Assistência de Enfermagem, Coleta de Amostras Sanguíneas, Conhecimentos, Atitudes e Prática em Saúde, Qualidade da Assistência à Saúde

## Abstract

**Objetivos:**

investigar os conhecimentos, as atitudes e as práticas dos enfermeiros sobre a colheita de hemoculturas.

**Métodos:**

estudo transversal, desenvolvido em cinco hospitais públicos brasileiros, com 112 enfermeiros. Os dados foram coletados por questionário adaptado e analisados por estatística descritiva e inferencial.

**Resultados:**

enfermeiros que não se julgaram capazes de coletar hemoculturas tiveram 72% menos chance de realizar a colheita no sítio recomendado e 83% menos chance de utilizar assertivamente a mesma agulha da punção para inocular o sangue nos frascos. Enfermeiros que trabalham no pronto atendimento médico tiveram 75% menos chance de saber o *benchmark* internacionalmente da taxa de contaminação de hemoculturas e aqueles com menos de 5 anos na função diminuem em 79% a chance de assertividade nesta questão.

**Conclusões:**

há lacunas nos conhecimentos, atitudes e práticas dos enfermeiros sobre a colheita de hemoculturas. Padronização da técnica, educação periódica, supervisão e orientação da equipe de coleta e auditoria dos processos são estratégias de enfrentamento recomendadas.

## INTRODUÇÃO

As hemoculturas são os testes laboratoriais mais utilizados na prática clínica para investigar suspeita de infecção da corrente sanguínea (ICS). O exame consiste na colheita e inoculação de uma amostra de sangue em frasco de hemocultura contendo condições ideais para o crescimento microbiano. O exame é importante para definir o patógeno causador da infecção e o seu perfil de sensibilidade aos antimicrobianos, a fim de gerenciar e tratar com segurança as ICS^(1,2)^.

O isolamento de microrganismos que não são patogênicos para o paciente, como aqueles provenientes da microbiota da pele, de equipamentos ou superfícies ambientais, das mãos do profissional que executa a colheita, configura-se como contaminação da amostra^(1,3)^. Com isso, resultados falso-positivos podem ser gerados e, por consequência, prejuízos ao paciente e ao estabelecimento de saúde, como aumento da permanência hospitalar, intervenções adicionais indevidas, sofrimento do paciente e familiar, surgimento de microrganismos multidroga resistentes (MMDR), uso desnecessário de recursos humanos e materiais e oneração do sistema de saúde^(4)^.

As taxas de contaminação de hemoculturas fornecem uma métrica importante acerca da qualidade dos serviços prestados e devem ser mantidas em níveis tão baixos quanto possível^(4)^. Contudo, vários estudos^(5,6,7,8,9)^ registram elevadas taxas de contaminação, revelando que manter as taxas de contaminação de hemoculturas abaixo do *benchmark* internacionalmente recomendado de 3% pela *American Society of Microbiology* permanece como velho desafio aos estabelecimentos de saúde^(10)^.

A colheita de hemocultura é uma etapa crítica e uma prática frequente no cotidiano da Enfermagem. A implementação de protocolos de colheita e educação da equipe contribui para a redução das taxas de contaminação^(11)^. Assim, diversas estratégias preventivas já foram testadas para a tomada de decisão e construção de protocolos cientificamente fundamentados: técnicas de antissepsia^(12)^, ocupação do profissional responsável pela colheita^(13,14)^, soluções antissépticas^(15,16,17)^, capacitação da equipe de colheita^(5)^, restringir a colheita de amostras a uma equipe treinada e dedicada para a finalidade^(9)^, dentre outras.

A literatura, entretanto, carece de estudos que avaliem o nível de conhecimento, as atitudes e as práticas dos profissionais que comumente colhem sangue para cultura: a equipe de enfermagem. Poucas pesquisas foram realizadas acerca da temática^(18,19)^, apenas uma com enfermeiros^(20)^, e todas apontam, além da lacuna de conhecimento, a necessidade de treinamento das equipes para prevenir práticas e resultados errôneos que podem influenciar na qualidade da assistência prestada.

## OBJETIVOS

Investigar os conhecimentos, as atitudes e as práticas dos enfermeiros sobre a colheita de hemoculturas.

## MÉTODOS

### Aspectos éticos

O estudo foi conduzido de acordo com as diretrizes de ética nacionais e internacionais e aprovado pelo Comitê de Ética em Pesquisa da Universidade Federal de Mato Grosso do Sul, cujo parecer está anexado à presente submissão. Os enfermeiros que aceitaram participar do estudo assinaram o TCLE.

### Desenho, local do estudo e período

Trata-se de um estudo transversal, desenvolvido por meio de um inquérito de Conhecimentos, Atitudes e Práticas (CAP), norteado pela ferramenta STROBE, realizado nos setores de Pronto Atendimento Médico (PAM) e Unidade de Terapia Intensiva (UTI) de cinco hospitais públicos de ensino e de grande porte do estado de Mato Grosso do Sul: três situados na capital Campo Grande e dois localizados no interior do estado. Os dados foram coletados mês a mês no primeiro semestre de 2022.

### População, critérios de inclusão e exclusão

Foram incluídos todos os enfermeiros lotados nas unidades investigadas e excluídos aqueles que estavam de licença ou férias e que se recusaram a responder o questionário por pelo menos três vezes consecutivas. O profissional que trabalhava em mais de uma instituição incluída neste estudo foi incluso apenas uma vez.

### Protocolo do estudo

Os dados foram coletados por meio de instrumento adaptado^(20)^ composto por 25 questões distribuídas em duas seções: (i) variáveis sociodemográficas e profissionais e (ii) componentes CAP. Este último incluiu nove questões objetivas, com respostas curtas (sim, não ou não sei; e opções pré-estabelecidas). O tempo médio de resposta do questionário foi de 10 minutos.

Com o intuito de averiguar a operacionalidade da pesquisa e se as questões ofereciam alguma dificuldade de interpretação aos participantes, antes do início da coleta de dados foi realizado um teste piloto com enfermeiros que atuavam na Unidade Coronariana de um dos hospitais. Após a realização do pré-teste, foi identificada a necessidade de ser incluída no questionário a seguinte questão: “A colheita de hemocultura é uma rotina do seu trabalho?”. Depois disso, nenhuma alteração foi realizada no instrumento.

Os profissionais que atenderam aos critérios de inclusão e concordaram em participar da pesquisa mediante leitura e assinatura do Termo de Consentimento Livre e Esclarecido (TCLE) foram convidados a participar do estudo na qualidade de voluntários. Os dados foram coletados *in loco* e foi vedada a consulta a qualquer fonte de informação – internet, livros, manuais, colegas de trabalho, entre outras. Coube ao pesquisador garantir o completo preenchimento do inquérito sem consulta. Visando obter dados fidedignos, não houve limite de tempo para o completo preenchimento do questionário.

### Análise dos resultados e estatística

Os dados foram analisados por estatística descritiva e inferencial no software estatístico livre R, versão 4.2.0. Para verificar a associação das variáveis sociodemográficas (sexo, idade, grau de instrução) e profissionais (tempo de experiência na profissão, tempo de atuação na unidade e turno de trabalho) com os componentes CAP, aplicou-se o Teste Qui-Quadrado ou Exato de Fisher. O *Odds Ratio* (OR), com intervalo de confiança (IC) de 95%, foi utilizado para estimar a magnitude das associações. O Teste de Kolmogorov-Smirnov foi empregado para verificar a normalidade dos dados. O nível de significância adotado foi de 5% para todos os testes estatísticos aplicados.

## RESULTADOS

Foram elegíveis para o estudo 194 enfermeiros, dos quais 82 (42,3%) foram excluídos: 23 (28%) por estarem de férias, licença ou afastados, 4 (4,9%) por se recusarem a participar do estudo e 55 (67,1%) por estarem ausentes de seus setores de trabalho ou se recusaram a responder o questionário (Figura 1). Portanto, a amostra foi constituída por 112 (57,7%) enfermeiros, cujas características estão apresentadas na Tabela 1.

**Figura 1:** Fluxograma de definição da amostra do estudo, Campo Grande, Mato Grosso do Sul, Brasil, 2022

**Tabela 1:** Características sociodemográficas e ocupacionais dos enfermeiros dos três hospitais da capital e dos dois hospitais do interior, (N=112), Campo Grande, Mato Grosso do Sul, Brasil, 2022

Variável	n	%
Sexo		
Feminino	83	74,11
Masculino	29	25,89
Idade, anos (M±DP)	35,84±7,27	
≤ 35	48	54,55
> 35	40	45,45
Formação		
Especialização em UTI ou PAM	71	63,38
Especialização (exceto UTI e PAM)	37	33,04
Mestrado	2	1,79
Doutorado	2	1,79
Hospital		
A	38	33,93
B	29	25,89
C	24	21,43
D	17	15,18
E	4	3,57
Setor		
PAM	45	40,18
UTI	67	59,82
Tempo de formação na enfermagem, anos (M±DP)	10±5,55	
≤ 10	60	53,57
> 10	52	46,43
Tempo na função que exerce, anos (M±DP)	6,50±5,33	
≤ 7	61	54,46
> 7	51	45,54
Tempo de exercício em UTI ou PAM, anos (M±DP)	4,50±4,46	
≤ 5	66	58,93
> 5	46	41,07
Turno de trabalho		
Matutino	39	34,82
Vespertino	29	25,89
Noturno	44	39,29
A colheita de hemocultura é uma rotina do seu trabalho?		
Não	42	37,50
Sim	70	62,50
Neste serviço, você recebeu treinamento para colheita de hemocultura?		
Não	55	49,11
Sim	53	47,32
Não lembra	4	3,57
Se julga capacitado para coletar adequadamente hemoculturas?		
Não	26	23,21
Sim	86	76,79
Gostaria de receber capacitação para coletar hemoculturas?		
Não	9	8,04
Sim	103	91,96
Qual é o profissional que geralmente coleta as amostras de sangue para os exames de hemocultura no seu setor?		
Enfermeiro	58	51,79
Técnico do laboratório	53	47,32
Técnico de enfermagem	1	0,89
O seu hospital tem um procedimento operacional padrão de colheita de sangue para cultura?		
Sim	64	57,14
Não sabe	48	42,86

*M – Média; DP – Desvio Padrão; UTI – Unidade de Terapia Intensiva; PAM – Pronto Atendimento Médico.*

A maioria dos enfermeiros é do sexo feminino (74,11%) e possui especialização em UTI ou PAM (63,38%). Em média, tem 10 anos de formação em enfermagem, com 53,57% tendo 10 anos ou menos. O tempo médio na função é de 6,50 anos, com 54,46% atuando por até 7 anos. A maioria trabalha em UTI (59,82%) por cerca de 4,50 anos. Quanto à colheita de hemocultura, 62,50% relatam que é rotina do trabalho, 47,32% já receberam treinamento para isso e 76,79% se consideram capacitados para coletar adequadamente o exame. Além disso, 91,96% expressam interesse em receber capacitação adicional. Sobre procedimentos operacionais padrão (POP’s), 57,14% dos enfermeiros relatam que seu hospital possui, enquanto 42,86% não têm essa informação disponível (Tabela 1).

Observou-se que os enfermeiros são os profissionais que mais executam as colheitas de hemocultura (51,8%). A maioria realiza uma nova punção vascular ao colher as amostras de sangue para cultura (68,7%) e utiliza álcool 70% tanto para a antissepsia do sítio de punção (74,1%) quanto para a desinfecção da parte superior dos frascos (89,3%).

Houve predominância da técnica limpa/asséptica para a coleta (68,7%) em comparação à estéril (29,5%), e o volume de 10 ml foi o mais assinalado (58,9%) pelos enfermeiros, apesar de 66,1% não saberem a porcentagem do frasco que corresponde ao volume ideal de sangue a ser coletado. A Tabela 2 apresenta as frequências de respostas dos componentes CAP.

**Tabela 2:** Distribuição das respostas segundo conhecimentos, atitudes e práticas dos enfermeiros dos três hospitais da capital e dos dois hospitais do interior sobre colheita de hemocultura, (N=112), Campo Grande, Mato Grosso do Sul, Brasil, 2022

Questões e respostas	n	%
Práticas		
De qual sítio você normalmente coleta sangue para hemocultura?		
Acesso venoso periférico previamente instalado	7	6,25
Acesso venoso central	26	23,21
Nova punção vascular	77	68,75
Diversos	2	1,79
Qual das seguintes soluções você utiliza para realizar antissepsia do sítio de punção para a colheita de sangue para cultura?	0	
Álcool 70%	83	74,11
Solução de Clorexidina	29	25,89
Qual das seguintes soluções você utiliza para desinfetar a parte superior dos frascos de hemocultura?		
Álcool 70%	100	89,29
Solução de Clorexidina	12	10,71
Atitudes		
Quantos mililitros de sangue você coleta para o exame de hemocultura?		
1ml	1	0,89
5ml	22	19,64
10ml	66	58,93
20ml	18	16,07
Outro	5	4,46
Qual técnica você utiliza para realizar a coleta das amostras?		
Técnica estéril	33	29,46
Técnica limpa/asséptica	77	68,75
Não sabe	2	1,79
Conhecimentos		
Qual das alternativas a seguir é a taxa de contaminação de hemoculturas do seu hospital?		
0-3%	4	3,57
3-5%	2	1,79
5-10%	5	4,46
>10%	2	1,79
Não sabe	99	88,39
Qual é o valor internacionalmente recomendado para a taxa de contaminação de hemoculturas?		
0%	4	3,57
<3%	18	16,07
<5%	9	8,04
<7%	1	0,89
Não sabe	80	71,43
O volume ideal de sangue a ser coletado corresponde a qual porcentagem do volume total do frasco de coleta?		
5%	2	1,79
10%	17	15,18
15%	1	0,89
20%	15	13,39
Não sabe	74	66,07
Outra resposta	3	2,68
A troca de agulhas entre a punção de coleta e a distribuição do sangue no frasco de hemocultura é recomendada?		
Sim	45	40,18
Não	31	27,68
Não sabe	36	32,14

As perguntas da Tabela 2 foram cruzadas com as variáveis sociodemográficas e ocupacionais dos participantes, cujas associações estatisticamente significativas são apresentadas na Tabela 3. Descobriu-se que enfermeiros que não se julgam capacitados para coletar hemoculturas tiveram 72% menos chances de realizar a colheita no sítio preconizado (OR: 0,28; IC 95%: 0,11-0,69) e 83% menos chances de usar assertivamente a mesma agulha da punção vascular para a inoculação de sangue no frasco de hemocultura (OR: 0,07; IC 95%: 0,01-0,58). Sobre o *benchmark* internacionalmente aceito de 3% ^(10)^ para a taxa de contaminação de hemocultura, a chance de possuir esse conhecimento por parte dos enfermeiros foi: (i) 75% menor para aqueles que trabalham no PAM em comparação aos seus homólogos de UTI (OR: 0,25; IC 95%: 0,07-0,91); e (ii) 79% menor para aqueles com menos de 5 anos na função em comparação com o grupo que trabalha há mais tempo (OR: 0,21; IC 95%: 0,07-0,63). Além disso, enfermeiros que atuam no turno matutino tiveram maior percentual de acerto sobre o referido *benchmark* quando comparados aos que atuam em outros turnos (p=0,008).

**Tabela 3:** Associação dos conhecimentos, atitudes e práticas dos enfermeiros a partir dos dados sociodemográficos e ocupacionais

Variável	Frequência (n) e percentagem (%) de acertos nas questões					
	Questão 1		Questão 2		Questão 3	
	n(%)	Valor de *p*	n(%)	Valor de *p*	n(%)	Valor de *p*
Setor						
PAM	29(64,4)	0,420	3(6,7)	0,026^†^	13(28,9)	0,815
UTI	48(71,6)		15(22,4)		18(26,9)	
Tempo de exercício em UTI ou PAM						
≤ 5 anos	43(65,1)	0,325	5(7,6)	0,003^‡^	15(22,7)	0,161
> 5 anos	34(73,9)		13(28,3)		16(34,8)	
Turno de trabalho						
Matutino	26(66,7)	0,629	12(30,8)	0,008	9(23,1)	0,674
Vespertino	22(75,9)		2(6,9)		8(27,6)	
Noturno	29(65,9)		4(9,1)		14(31,8)	
Se julga capacitado para coletar adequadamente hemoculturas?						
Não	12(46,2)	0,005*	2(7,7)	0,235	1(3,8)	0,002^§^
Sim	65(75,6)		16(18,6)		30(34,9)	

Questão 1: “Qual sítio você normalmente coleta sangue para hemocultura?”; Questão 2: “Qual é o valor internacionalmente recomendado para a taxa de contaminação de hemoculturas?”; Questão 3: “A troca de agulhas entre a punção de coleta e a distribuição do sangue no frasco de hemocultura é recomendada?”; *OR: 0,28 [IC 95%: 0,11-0,69]; ^†^OR: 0,25 [IC 95%: 0,07-0,91]; ^‡^OR: 0,21[IC 95%: 0,07-0,63]; ^§^OR: 0,07 [IC 95%: 0,01-0,58].

## DISCUSSÃO

Este é o primeiro estudo sobre CAP de enfermeiros que atuam em UTI e PAM sobre colheita de hemocultura no Brasil. Seus resultados contribuem para o avanço nas fronteiras do conhecimento, pois fornecem informações sobre o que enfermeiros de hospitais de ensino de grande porte sabem acerca da colheita de hemocultura, bem como suas atitudes e práticas. A partir da observação da prática clínica, é possível inferir que o enfermeiro desempenha um papel crucial na colheita de amostras de hemocultura, pois além de executar as competências técnicas e científicas exigidas pelo procedimento e ser o responsável por realizá-lo em contexto hospitalar, também é responsável por garantir a segurança e as boas práticas inerentes à colheita das amostras.

### Conhecimentos sobre colheita de hemocultura

Apesar de se tratarem de hospitais de ensino, identificou-se carência de conhecimentos dos profissionais acerca do procedimento de colheita de hemoculturas, o que pode ser explicado pela insuficiência de treinamento e capacitação dos profissionais para a realização do procedimento, visto que grande parte (49,1%) não recebeu treinamento no local de trabalho. Conforme a Organização Mundial da Saúde, educação e treinamento são necessários para todo o pessoal que colhe amostras sanguíneas, devendo incluir compreensão da anatomia, percepção dos riscos de exposição ao sangue e das consequências da deficiência na prevenção e controle de infecções^(21)^.

A maioria dos enfermeiros não soube a taxa de contaminação de hemoculturas do hospital em que trabalha (81,4%), nem o *benchmark* internacionalmente recomendado para contaminação de hemoculturas (71,4%) ou a porcentagem do volume total do frasco correspondente ao volume de sangue ideal a ser coletado (66,1%). Esse achado é possivelmente sustentado no déficit de treinamento realizado pelos profissionais e na falta de monitoramento dos indicadores assistenciais.

Implementar estratégias de treinamento para a equipe de colheita gera custos imediatos às instituições devido à necessidade de educação continuada e investimento em capacitação da equipe, o que justifica boa parte dos enfermeiros não ter recebido treinamento no local de trabalho. Todavia, os benefícios gerados com a qualificação e capacitação das equipes diminuem custos futuros, que podem ser ainda mais onerosos do que aqueles dispendidos com treinamento.

Revelou-se que os enfermeiros que trabalham em UTI e que têm mais de 5 anos de exercício obtiveram maior assertividade sobre o valor internacionalmente aceito de 3% para a taxa de contaminação de hemocultura. Este achado pode ser justificado pelo fato da UTI ser um setor cujas infecções hospitalares são mais preocupantes e debatidas. Enfermeiros com mais tempo de trabalho já foram expostos a mais situações em que tiveram que coletar sangue para hemocultura e também têm mais contato com as políticas e *bundles* de prevenção de infecção hospitalar – como Procedimento Operacional Padrão (POP) –, apesar de 43% dos profissionais não saberem da existência de um POP sobre colheita de hemocultura em seus hospitais.

Enfermeiros que trabalham no turno matutino tiveram maior percentual de acerto quando comparados aos dos outros turnos. Normalmente, a colheita de amostras para exames laboratoriais ocorre majoritariamente no período matutino. Dessa forma, os profissionais que trabalham pela manhã são mais habituados a colher material biológico para exames, incluindo hemocultura. Como esses enfermeiros realizam mais vezes o procedimento, acabam aprimorando a técnica de colheita e incorporando os conhecimentos das principais diretrizes, o que pode justificar sua maior assertividade.

Profissionais que se julgam capacitados para realizar a colheita das amostras tiveram maiores porcentagens de acertos quando indagados sobre o sítio de punção recomendado e a necessidade da troca de agulha entre a punção e a inoculação do sangue nos frascos de hemocultura. Infere-se que enfermeiros que se julgam capacitados, de fato, têm mais conhecimento, o que gera autoconfiança e segurança. A autoconfiança, embora não seja um fator determinante para o conhecimento, é um importante atributo para a assertividade técnico-operacional. Um estudo realizado com graduandos em enfermagem na Turquia mostrou que assertividade e autoconfiança estão intrinsecamente associados^(22)^. Esses achados ressaltam o papel central do conhecimento nos indicadores de saúde.

### Atitudes sobre colheita de hemocultura

A técnica mais comumente utilizada pelos enfermeiros para a colheita das amostras foi a asséptica (68,7%), justificada pela sua praticidade, baixo custo e preferência dos profissionais. Um ensaio clínico^(11)^ concluiu que mais importante do que a técnica utilizada – asséptica ou estéril – é o rigor asséptico na obtenção das amostras, assim como a aplicação de protocolo de colheita e a qualificação dos profissionais que a realizam. Neste estudo CAP, evidenciou-se que a maioria dos enfermeiros (57,1%) tem conhecimento do POP da sua instituição, mas carece de capacitação e qualificação.

No que diz respeito à atitude dos enfermeiros durante a colheita de sangue para cultura, 58,9% aspiram 10ml para cada frasco de hemocultura. Tal atitude pode estar ancorada na padronização das instituições acerca do procedimento, visto que a maioria dos profissionais alega ter conhecimento do POP do local em que trabalha. A Agência Nacional de Vigilância Sanitária (Anvisa) recomenda que a quantidade de sangue inoculada em cada frasco seja de 10ml e defende que a colheita do valor correto aumenta em 15% a chance de isolamento do patógeno^(23)^.

### Práticas sobre colheita de hemocultura

A maioria dos enfermeiros (68,7%) realiza uma nova punção vascular, arterial ou venosa, para a colheita das amostras. Entretanto, uma parcela considerável de profissionais ainda obtém o sangue de cateteres previamente instalados, centrais (23,2%) e periféricos (6,2%). Tal prática se justifica pela facilidade na obtenção das amostras, visto que uma nova punção vascular demanda mais tempo do profissional. Além disso, muitas vezes há fatores que dificultam essa nova punção, como a condição clínica, estado nutricional e escassez de rede vascular acessível, entre outros, que se acentuam ainda mais em pacientes mais graves, que representam o perfil majoritário dos pacientes atendidos pelos enfermeiros participantes deste estudo.

A solução mais utilizada tanto para a antissepsia do sítio de punção (74,1%) quanto para a desinfecção da borracha dos frascos de hemocultura (89,3%) foi o álcool 70%. Além de eficiente, este antisséptico é menos oneroso quando comparado a outros comumente utilizados em hospitais, como a clorexidina alcoólica, o que provavelmente explica sua disponibilidade nas instituições. Um Ensaio Clínico Randomizado realizado em um centro médico universitário em Israel mostrou que tanto o álcool 70% quanto soluções alcoólicas com gluconato de clorexidina 2% e soluções não alcoólicas foram capazes de reduzir a contaminação de hemoculturas^(15)^. No entanto, uma revisão sistemática com meta-análise demonstrou que os produtos que contêm álcool são superiores a produtos sem álcool para a colheita de hemocultura devido à ação antisséptica imediata do álcool e sua secagem rápida em até 30 segundos^(16)^. O uso de antissépticos ainda é divergente na literatura e carece de um consenso sobre qual solução é mais eficaz na redução das taxas de contaminação.

### Limitações do estudo

A baixa adesão dos profissionais para responder ao questionário e a sua aplicação durante a rotina de serviço podem ter limitado os resultados encontrados, considerando que os enfermeiros responderam ao mesmo durante seus turnos de serviço. Outra limitação a ser considerada é a escassez de literatura e a falta de estudos com a metodologia CAP sobre a temática abordada. Por fim, a natureza transversal do estudo não permite estabelecer relações de causa e efeito entre as variáveis investigadas.

### Contribuições para a Enfermagem e/ou Saúde

Este estudo não só fornece informações valiosas sobre a prática da coleta de hemoculturas, mas também oferece contribuições significativas para a enfermagem e a saúde pública. Ao identificar pontos críticos e lacunas de conhecimento entre os enfermeiros, este estudo aponta áreas específicas que requerem intervenção educacional e programas de melhoria da qualidade. A implementação de estratégias educativas e de treinamento baseadas nos resultados deste estudo poderá resultar em melhorias significativas nas práticas de coleta de hemoculturas, contribuindo para a redução da contaminação de amostras e minimização de resultados falsos positivos.

## CONCLUSÕES

Os resultados dessa pesquisa evidenciam a predominância de flebotomistas enfermeiros, do álcool 70% entre os antissépticos e da técnica asséptica em detrimento da estéril. Houve deficiências nos conhecimentos, atitudes e práticas dos enfermeiros para colher com segurança amostras de hemoculturas. Enfermeiros que não se julgam capacitados para colher hemocultura e com menos de cinco anos na função tiveram associações desfavoráveis nas questões de CAP. O enfrentamento dessas adversidades inclui treinamento e capacitação frequentes das equipes por meio da educação permanente, além da implementação de protocolos padronizados de colheita. Faz-se necessário a vigilância de processos e resultados, além de constante supervisão, orientação e auditoria dos profissionais quanto à realização correta do procedimento. Pesquisas futuras poderão avaliar o impacto dessas intervenções.
